# A Genetic Variant in Primary miR-378 Is Associated with Risk and Prognosis of Hepatocellular Carcinoma in a Chinese Population

**DOI:** 10.1371/journal.pone.0093707

**Published:** 2014-04-21

**Authors:** Jiaze An, Jibin Liu, Li Liu, Yao Liu, Yun Pan, Mingde Huang, Fuzhen Qi, Juan Wen, Kaipeng Xie, Hongxia Ma, Hongbing Shen, Zhibin Hu

**Affiliations:** 1 Department of Hepatobiliary Surgery, Xijing Hospital, Fourth Military Medical University, Xi’an, China; 2 Department of Hepatobiliary Surgery, Nantong Tumor Hospital, Nantong, Nantong, Jiangsu, China; 3 Digestive Endoscopy Center, the First Affiliated Hospital of Nanjing Medical University, Nanjing, Jiangsu, China; 4 Pathology Center and Department of Pathology, Soochow University, Suzhou, Jiangsu, China; 5 Department of Epidemiology and Biostatistics, Jiangsu Key Lab of Cancer Biomarkers, Prevention and Treatment, Cancer Center, School of Public Health, Nanjing Medical University, Nanjing, Jiangsu, China; 6 Department of Oncology, Huai’an First People’s Hospital, Nanjing Medical University, Huai’an, Jiangsu, China; 7 Department of Hepatopancreatobiliary Surgery, Huai’an First People’s Hospital, Nanjing Medical University, Huai’an, Jiangsu, China; 8 State Key Laboratory of Reproductive Medicine, Nanjing Medical University, Nanjing, Jiangsu, China; MOE Key Laboratory of Environment and Health, School of Public Health, Tongji Medical College, Huazhong University of Science and Technology, China

## Abstract

**Background:**

MiR-378 has been reported to be related to cell survival, tumor growth and angiogenesis and may participate in hepatocellular carcinoma (HCC) development and prognosis. Genetic variants in primary miR-378 (pri-miR-378) may impact miR-378 expression and contribute to HCC risk and survival. This study aimed to assess the associations between a genetic variant in primary miR-378 and HCC susceptibility and prognosis.

**Methods:**

We conducted a case-control study to analyze the association of rs1076064 in pri-miR-378 with hepatocellular carcinoma risk in 1300 HCC patients with positive hepatitis B virus (HBV) and 1344 HBV carriers. Then, we evaluated the correlation between the polymorphism and hepatocellular carcinoma prognosis in 331 HCC patients at either intermediate or advanced stage without surgical treatment.

**Results:**

The variant genotypes of rs1076064 were associated with a decreased HCC risk in HBV carriers [Adjusted odds ratio (OR) = 0.90, 95% confidence intervals (CI) = 0.81–1.00, *P = *0.047]. Moreover, HCC patients with the variant genotypes were associated with a better survival [Adjusted hazard ratio (HR) = 0.70, 95% CIs = 0.59–0.83, *P*<0.0001 in an additive genetic model]. The reporter gene assay showed that the variant G allele of rs1076064 exerted higher promoter activity than the A allele.

**Conclusions:**

These findings indicate that rs1076064 may be a biomarker for HCC susceptibility and prognosis through altering pri-miR-378 transcription.

## Introduction

Hepatocellular carcinoma (HCC) is a major cancer burden in China [1]. Hepatitis B virus (HBV) infection has been well established as a major risk factor in HCC carcinogenesis [2]. Hereditary factors may also play critical roles in the pathogenesis of HCC, together with other environmental factors, such as alcohol drinking and aflatoxins ingestion [3], [4]. Surgical resection and liver transplantation may cure HCC, but about 85% of patients have locally advanced tumor or distant metastasis at the time of diagnosis, and are not suitable candidates for surgery [5]. Previous studies have demonstrated that genetic factors play important roles in the progression of HCC, including chemosensitivity, tumor recurrence and prognosis [6]–[10].

MicroRNAs (miRNA) are a class of small non-coding RNAs exerting post-translational regulation through pairing target mRNAs. MiRNAs may act as oncogenes or tumor suppressor genes depending on their downstream target genes. Alterations of miRNAs, including expression disorders and mutations, are involved in the initiation and progression of human cancers [11]. Our group previously reported that single nucleotide polymorphisms (SNPs) in precursor miRNA (pre-miRNA) or primary miRNA (pri-miRNA), may influence survival and susceptibility of human cancers including HCC [12]–[14].

Recent studies revealed a subset of miRNAs dysregulated in HCC, and could be used as biomarkers for early detection and prognosis prediction [15]–[18]. MiR-378 has been reported to be related to cell survival, tumor growth and angiogenesis [19], [20]. Song *et al* reported that miR-378 inhibit hepatocyte proliferation during liver regeneration [21]. Besides, accumulating data identified miR-378 was down-regulated in several cancers compared with that in para-non-tumor tissues [22]–[26]. In this study, we hypothesized that SNP rs1076064 in pri-miR-378 may contribute to HCC in both cancer development and survival.

## Materials and Methods

### Study Population

This study was approved by the institutional review board of Nanjing Medical University. Written informed consent was obtained from every subject. The subjects’ enrollment was described previously [27], [28]. All cancer patients were confirmed by pathological examination and/or α-fetoprotein elevation (>400 ng/ml) combined with imaging examination (Magnetic resonance imaging, MRI and/or computerized tomography, CT). Eventually, 1300 HBV positive (hepatitis C virus, HCV, negative) HCC cases consented to participate in the study. The controls were positive for both HBV surface antigen (HBsAg) and antibody to hepatitis B core antigen (anti-HBc), negative for HCV antibody (anti-HCV), and matched to the HCC cases on age and sex. These selected controls had no self-reported history of cancer.

In consideration of prognostic modeling in HCC patients has a high complexity and should consider four tightly related aspects: tumor stage, degree of liver function impairment, patient’s general condition, and treatment efficacy, we use the Barcelona Clinic Liver Cancer (BCLC) Stage System which is a good stage system in evaluating the prognosis of HCC [29].To construct a relatively homogenous population with similar treatment, our study was restricted to HCC patients in intermediate stage (B) or advanced stage(C) without surgery to analyze the prognosis. We recruited 414 intermediate or advanced HCC patients from Nantong Tumor Hospital and the First Affiliated Hospital of Nanjing Medical University, Jiangsu, China (see **Table S1 in** **File S1**). All patients were followed up prospectively every 3 months from the time of enrollment by personal or family contacts until death or last time of follow-up. As a result, a total of 331 HCC patients who had complete follow-ups and clinical information were enrolled in our study with the response rate as 80.0%. The maximum follow-up time (MFT) for the 331 patients involved in the present study was 60.7 months (last follow-up in January 2013) and the median survival time (MST) was 14.5 months.

### Serological Testing

HBsAg, anti-HBs, anti-HBc and anti-HCV were detected by the enzyme-linked immunosorbent assay (Kehua Bio-engineering Co., Ltd., Shanghai, China) following the manufacturer’s instructions as described previously [27].

### SNPs Selection and Genotyping

Based on the HapMap database, we found two SNPs, rs1076064 and rs1076063 in pri-miR-378, with minor allele frequency (MAF) in Han Chines population >0.05. The two SNPs were in high linkage disequilibrium (LD) (r^2^ = 1) and in a LD block. Thus, we genotyped only one SNP, rs1076064.

Genomic DNA was extracted from a leukocyte pellet by traditional proteinase K digestion, phenol-chloroform extraction and ethanol precipitation. The SNP, rs1076064 A>G was genotyped using the TaqMan allelic discrimination assay on a 7900 system (Applied Biosystems). The primers and probes for rs1076064 were as follows. Primers: sense, 5′-TCCTATCAATTACATTTCCCAAGTTG, antisense, 5′-TGAAAGTTAATCTGGGACATTTGCT; Probes: allele A, FAM-TTAACCACATGACAGGC-MGB, allele G, HEX-TAACCACGTGACAGGC-MGB. The genotyping was performed blindly without knowing the subjects’ case or control status. Two blank (water) controls in each 384-well plate were used for quality control and more than 5% samples were randomly selected and repeated, yielding a 100% concordant. The success rate of genotyping for rs1076064 was above 99%.

### Construction of Luciferase Reporter Plasmids

**Figure 1:**
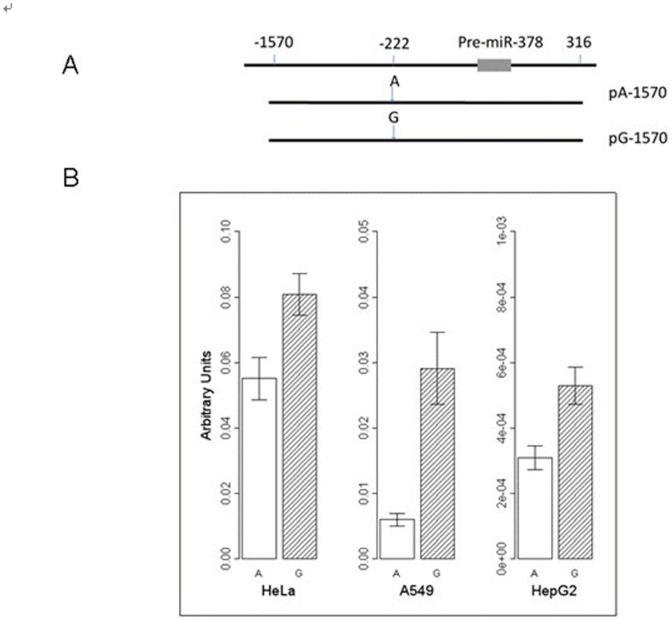
Promoter activity with different constructs containing different alleles of rs1076064 (A>G). (A) Schematic representation of the pre-miR-378 flanking region and reporter gene constructs. (B) Luciferase expression of constructs containing rs1076064 A or G allele in HeLa, A549, and HepG2 cells. All constructs were cotransfected with pRL-SV40 to standardize the transfection efficiency. Luciferase levels of the constructs, pGL3-Promoter and pRL-SV40 plasmids were determined in triplicate. Fold increase was measured by defining the activity of the empty pGL3-Promoter vector as 1. *Columns,* means from two independent experiments, each in triplicate; *bars,* SE.

The fragment (rs1076064 AA) corresponding to pre-miR-378 5′-flanking region was generated by PCR and cloned into the pGL3-promoter vector (Promega) with *Kpn I* and *Nhe I* digestions (Fig. 1A). The primers were: sense, 5′- TACGGTACCGAAGCTGGTGGCTTGGAGAC −3′; antisense, 5′- CAAGCTAGCCCCTGGATTAGCCACCAAAG −3′. The resultant plasmids were designated as pA-1570. The pA-1570 construct was then site-specifically mutated to create the constructs pG-1570, which contains −222G (rs1076064 G allele). All insertions were sequenced to verify the accuracy.

### Transient Transfections and Luciferase Assays

We seeded 5×10^5^ hepatocellular carcinoma cells, HepG2, human cervical cancer cells, HeLa, and human lung adenocarcinoma cells, A549, respectively. The plasmids pGL3- promoter and pGL3- promoter constructs with pre-miR-378 5′-flanking region containing different rs1076064 alleles were co-transfected with pRL-SV40 respectively. All transfections were carried out in triplicate. After 36 hours of incubation, cells were collected and analyzed for luciferase activity with the Dual-Luciferase Reporter Assay System (Promega).

### Statistical Analysis

The Student’s t-test and χ^2^ test were used to detect differences of demographic characteristics, genotype frequencies of the SNP between the cases and controls for continuous variables and categorical variables, respectively. Hardy–Weinberg equilibrium was assessed within patients by using a goodness-of-fit χ^2^ test. Associations between the genotypes and risk of HCC were estimated by computing odds ratios (ORs) and their 95% confidence intervals (CIs) from logistic regression analyses. Median survival time (MST) was calculated, and mean survival time was presented when the MST could not be calculated. Kaplan–Meier method and log-rank test were used to compare the survival time in different subgroups categorized by patient characteristics, clinical features and genotypes. Univariate and multivariate Cox proportional hazard regression analysis were performed to estimate the crude or adjusted hazard ratio (HR) and their 95% confidence intervals (CIs), with adjustment of age, gender, smoking status, drinking status, the Barcelona Clinic Liver Cancer (BCLC) staging system [29], and chemotherapy or TACE (transcatheter hepatic arterial chemoembolization) status. Cox stepwise regression model was also conducted to determine predictive factors to HCC prognosis, with a significance level of 0.050 for entering and 0.051 for removing the respective explanatory variables. The Chi-square-based *Q* test was applied to test the heterogeneity of associations between subgroups. Analyses were carried out using Statistical Analysis System software (version 9.1.3;SAS Institute, Cary, NC). All tests were two-sided and the criterion of statistical significance was set at *P*<0.05.

## Results

The demographic characteristics of the 1300 HCC cases with positive HBV, 1344 HBV carriers and 331 intermediate or advanced HCC cases were summarized in **Table S1 in** **File S1**. No significant difference was detected in the distributions of age, gender and smoking rates between the two groups. However, the drinking rates were higher among cases than that in controls. For the 331 HCC patients in stage B or C retained in our survival analysis, 258 died from HCC, and 2 died from other causes during a period up to 60.7 months of follow-up. For disease-specific survival analysis, the latter were considered as censored data in the analyses. Drinking and chemotherapy or TACE (transcatheter hepatic arterial chemoembolization) status were significantly associated with survival time of HCC (see **Table S1 in** **File S1**).

The genotype distribution of rs1076064 in HCC cases with positive HBV and HBV carriers is shown in **Table 1**. The successful genotyping rate for rs1076064 was 99.25%. Compared with the wild-type AA of rs1076064, those with AG and GG genotypes had a decreased risk of HCC with adjusted ORs of 0.93 (95% CIs = 0.77–1.11) and 0.81 (95% CIs = 0.65–1.00), respectively, and 0.90 (95% CIs = 0.81–1.00, *P* = 0.047) in an additive genetic model.

**Table 1:** Genotype frequencies of rs1076064 and HBV persistent infection and HCC susceptibility.

Genotype	HCC patients	HBV persistent carriers	OR (95%CIs)*	*P* *
	(N = 1300)	(N = 1344)		
**rs1076064**	n%	n%		
**AA**	378(29.7)	355(26.7)	1	
**AG**	598(46.3)	610(45.8)	0.93(0.77–1.11)	
**GG**	315(24.4)	367(27.6)	0.81(0.65–1.00)	
**Additive**			0.90(0.81–1.00)	0.047

CI, confidence interval; HBV, hepatitis B virus; HCC, hepatocellular carcinoma; OR, odds ratio.

*Adjusted for age, gender, smoking status and drinking status (HCC patients *vs.* HBV persistent carriers).

**Figure 2:**
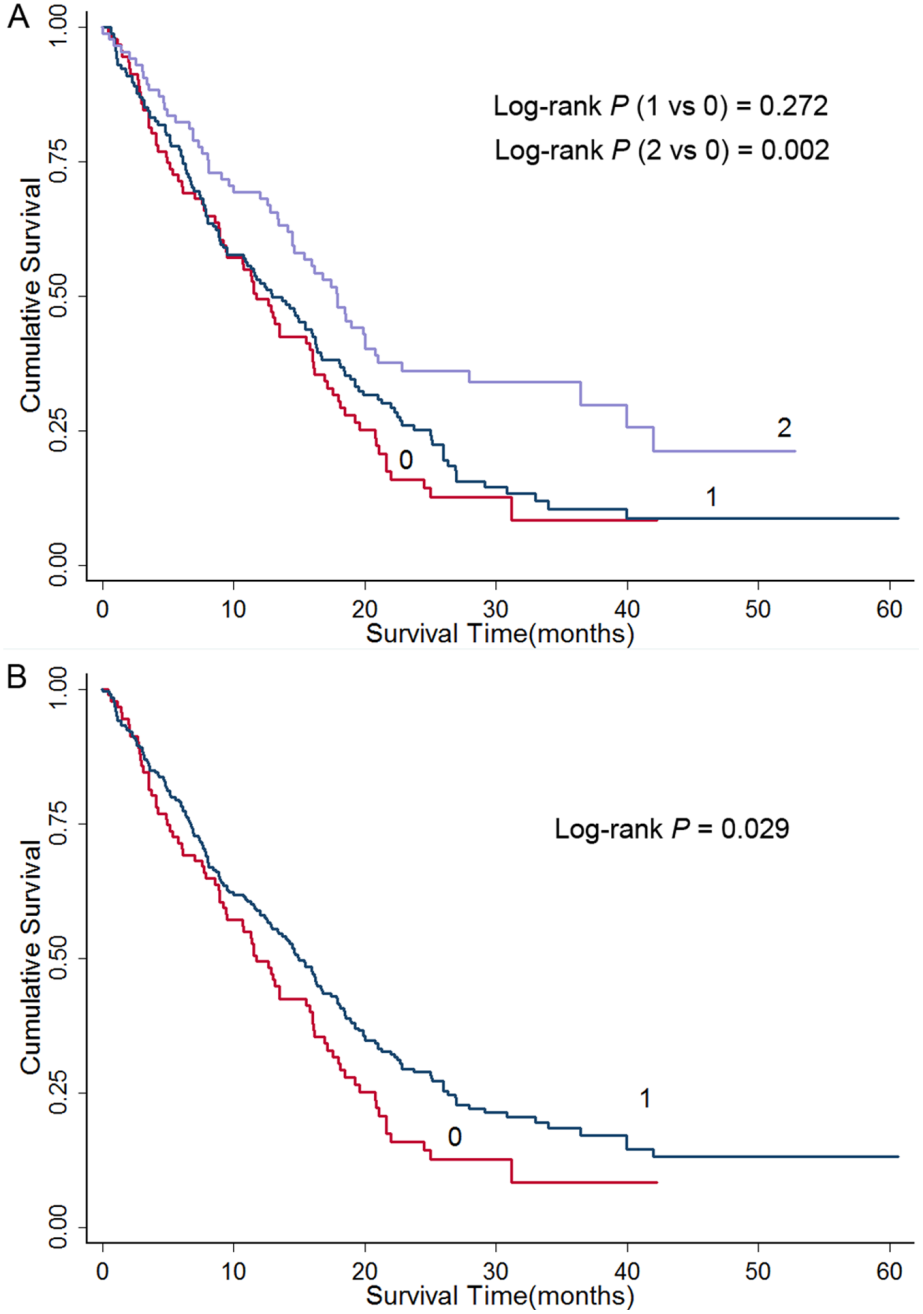
Kaplan-Meier plots of survival by miR-378 rs1076064 genotypes in HCC-specific survival. (A) “0” denotes patients with common genotype (AA); “1” denotes patients with AG; “2” denotes patients with GG; (B) “0” denotes patients with common genotypes (AA); “1” denotes those with variant genotypes (AG/GG).

Kaplan–Meier method and log-rank test were used to examine the associations of the rs1076064 variant with HCC survival. Log-rank test revealed that variant genotype GG of miR-378 rs1076064 had a significantly improved survival (MST, 17.9 months; Log-rank *P*: 0.002) compared with the homozygote AA (MST, 11.8 months; **Table 2**). Similarly, the patients carrying rs1076064 AG genotype demonstrated a longer survival as well (MST, 13.0 months), although not reaching statistical significance (Log-rank *P*: 0.272 for AG vs. AA). As shown in **Table 2**, the variant genotypes of rs1076064 were significantly associated with a better survival of HCC after adjusting for age, gender, smoking status, drinking status, BCLC stage, and chemotherapy or TACE status (additive model: adjusted HR = 0.70, 95% CI  = 0.59–0.83). Kaplan–Meier plots of HCC-specific survival grouped by rs1076064 genotypes were shown in **Figure 2**. Moreover, we conducted stepwise multivariate analyses with selected demographic characteristics, clinical features and rs1076064 genotypes on HCC survival. Four variables (age, drinking status, chemotherapy or TACE status and rs1076064 genotype in an additive model) entered the regression model with a significance level of 0.050 for entering and 0.051 for removing a variable (**Table 3**). When gender, smoking status, and BCLC stage were included in the final model, the effect of rs1076064 genotype was a significant prognostic factor for the survival of HCC patients (adjusted HR = 0.70, 95%CI = 0.59–0.84 in an additive model) (**Table 3**).

**Table 2:** Genotypes of miR378 polymorphisms and HCC survival.

Genotype	Total	Deaths	MST(Months)	Log-rank *P*	Crude HR(95% CI)	Adjusted HR(95% CI)*
**rs1076064(A>G)**	N = 330	N = 257				
**AA**	91	75	11.8		1	1
**AG**	154	126	13.0	0.272	0.85(0.64–1.14)	0.78(0.58–1.05)
**GG**	85	56	17.9	0.002	0.57(0.40–0.81)	0.48(0.33–0.69)
**Additive model**				0.003	0.75(0.64–0.89)	0.70(0.59–0.83)

MST, median survival time; HR, hazard ratio; CI, confidence interval; HCC, hepatocellular carcinoma; TACE, transcatheter hepatic arterial chemoembolization.

*Adjusted for age, gender, smoking status, drinking status, BCLC stage, and chemotherapy or TACE status.

**Table 3:** Multivariate Cox regression analysis on HCC survival.

Variables	β*	SE†	HR	95% CI	*P*
***Stepwise regression analysis***					
Chemotherapy or TACE (yes vs none)	−1.1741	0.1516	0.31	0.23–0.42	<0.0001
rs1076064 (additive model)	−0.3455	0.0874	0.71	0.60–0.84	0.0002
Age (>53 vs < = 53)	−0.4483	0.1353	0.64	0.49–0.83	0.0013
Drinking status (yes vs no)	0.3956	0.1312	1.49	1.15–1.92	0.0024
***Final regression analysis***					
Chemotherapy or TACE (yes vs none)	−1.198	0.1547	0.3	0.22–0.41	<0.0001
rs1076064 (additive model)	−0.3533	0.088	0.7	0.59–0.84	<0.0001
Drinking status (yes vs no)	0.5373	0.1695	1.71	1.23–2.39	0.0015
Age (>53 vs < = 53)	−0.4626	0.1371	0.63	0.48–0.82	0.0007

HR, hazard ratio; CI, confidence interval; HCC, hepatocellular carcinoma; BCLC stage, Barcelona Clinic Liver Cancer stage; TACE, transcatheter hepatic arterial chemoembolization.

*β is the estimated parameter of the regression model.

† SE is the standard error of the regression model.

The associations between miR-378 rs1076064 polymorphism and HCC susceptibility and survival were further evaluated by stratified analysis (see **Table S2&S3 in** **File S1**). No significant heterogeneity was detected between the subgroups for the association analyses of rs1076064 polymorphism and HCC susceptibility. However, we found that the rs1076064 variant genotypes were associated with a significantly better survival in males (adjusted HR = 0.65, 95% CI, 0.54–0.78). Although the heterogeneity of gender and rs1076064 genotypes was statistically significant (*P* = 0.032), no significant interactions were observed.

Given the SNP rs1076064 located in pri-miR-378, which is 222-bp upstream from pre-miR-378, it might influence miR-378 expression, and we examined the impact of rs1076064 on miR-378 expression by reporter gene analysis (**Figure 1A**). As shown in **Figure** **1B**, the construct pA-1570 containing the A allele of rs1076064 drove a significantly lower reporter gene expression compared with the G allele (HepG2 cells: A allele, 3.09×10^−4^±1.11×10^−4^, G allele, 5.29×10^−4^±1.69×10^−4^, *P* = 0.006; HeLa cells: A allele, 0.055±0.011, G allele, 0.081±0.011, *P* = 0.045; A549 cells: A allele, 0.006±0.002, G allele, 0.029±0.013, *P* = 0.008), suggesting the variant G allele of rs1076064 had higher promoter activity than the A allele.

## Discussion

In this study, we investigated the association of rs1076064 in pri-miR-378 with HCC risk and prognosis in a Chinese population. We found that the A to G base change of rs1076064 demonstrated protective effect on HCC risk. Moreover, our results showed that the effect of variant genotypes of rs1076064 was a significant favorable prognostic factor for intermediate or advanced stage HCC patients. Furthermore, we conduct reporter gene assay revealed that the G allele may exert higher promoter activity.

Accumulating data support miR-378 as a tumor suppressor. Guo *et al* and Yao *et al* independently found that miR-378 was down-regulated in gastric cancer tissues compared with that in normal gastric tissues [23], [26]. Wang *et al* identified that the expression level of miR-378 was lower in colonic cancer tissues compared with that in the para-cancerous controls [25]. Similar findings were also reported in oral and laryngeal cancers [22], [24]. Besides, Song *et al* reported that miR-378 inhibit hepatocyte proliferation during liver regeneration by performing partial hepatectomy on mice [21]. Controversial findings were found in breast cancer, in which miR-378 acted as a molecular switch involved in the orchestration of the Warburg effect and ledto cell proliferation [30]. In addition, *CYP2E1*, a pivotal cytochrome P450 isoform, has been indicated as a target of miR-378 and the amount of CYP2E1 protein was mainly regulated by miR-378 [31]. Considerable studies have reported that *CYP2E1* was primarily expressed in the liver tissue and not only involved in metabolism of drug but also enabled a lot of precarcinogens and prepoisons. Furthermore, genetic variants in *CYP2E1* gene were associated with malignancies of different origins including liver [32]–[35]. Interestingly, an insertion/deletion polymorphism in *IL-1* was identified to be associated with HCC susceptibility through disrupting miR-378 binding [36].

Although miR-378 may be a tumor suppressor in HCC, the genetic association between rs1076064 in pri-miR-378 and HCC risk was modest in HBV carriers. According to the web-based analysis tool of SNP, TFSEARCH 1.3, the A allele of rs1076064 may have affinity with transcription factors including AML-1a, Myc, USF, and C/EBP, while the G allele may have affinity with USF, Myc, Max, and SREBP-1. Among these potential transcription factors, AML-1a, SREBP-1 and C/EBP are transcriptional activators [37]–[39], while Max may be transcriptional activator or repressor when combined with Myc or Mad, respectively [40], [41]. Thus, we speculated that the variant genotypes of rs1076064 may impact the transcription of miR-378. In this study, we identified the G allele may exert higher promoter activity in several cancer cell lines using the reporter gene assay, compared to A allele.

To date, few studies have focused on the association between miR-378 polymorphisms and cancer development and prognosis. Remarkably, our group previously reported that miR-378 rs1076064 was associated with non–small cell lung cancer (NSCLC) survival in the screening set analysis, though it was not be corroborated in the validation set [13]. Our study further revealed that G allele of miR-378 rs1076064 was associated with a better survival in HCC.

There are a number of strengths in our study. To our knowledge, it is the first study to investigate the effect of miR-378 polymorphisms on the prognosis of HCC. Additionally, the population in this study was relatively homogeneous, in which all of the participants are at intermediate or advanced stage and without surgery. Finally, the stage system used in our study is the BCLC stage system which links staging with treatment modalities and with an estimation of life expectancy that is based on published response rates to the different treatments [42], and thereby own a great prognostic value in our survival analysis.

In spite of showing the rs1076064 polymorphism associated with the HCC development and prognosis, our study should be considered as preliminary and larger population based prospective studies are warranted to further elucidate the impact of rs1076064 on HCC susceptibility and prognosis.

## Supporting Information

File S1 **Supporting Information. Table S1.** Patient characteristics and clinical features. **Table S2.** Stratified analyses on association between rs1076064 and risk of HCC. **Table S3.** Stratified analysis of rs1076064 genotypes associated with HCC survival.
(DOC)
