# Supplementary material for: A Genetic Variant in Primary miR-378 Is Associated with Risk and Prognosis of Hepatocellular Carcinoma in a Chinese Population
Source: PLoS One. 2014 Apr 21;9(4):e93707. doi: 10.1371/journal.pone.0093707 (PMC3994025; doi:10.1371/journal.pone.0093707)
Supplement: File S1 — Supporting Information. Table S1. Patient characteristics and clinical features. Table S2. Stratified analyses on association between rs1076064 and risk of HCC. Table S3. Stratified analysis of rs1076064 genotypes associated with HCC survival. (DOC) [file pone.0093707.s001.doc]

Table S1. Patient characteristics and clinical features

| **Variables** | **HCC patients** | **HBV carriers** | ***P*** | **Advanced HCC patients ^a^** | | **MST (mo)** | **Log-rank *P*** | **HR(95% CI)** |
| --- | --- | --- | --- | --- | --- | --- | --- | --- |
|  | (N=1300)(%) | (N=1344)(%) |  | Patients (N=331)(%) | Deaths(N=258) |  |  |  |
| **Age,year(mean±SD)** | 52.9±10.5 | 52.7±11.1 | 0.839 | 53.2±10.9 | 52.6±11.0 | -- | -- | -- |
| **Age** |  |  |  |  |  |  | 0.257 |  |
| <=53 | -- | -- | -- | 173(52.3) | 138 | 14.3 |  | 1 |
| >53 | -- | -- |  | 158(47.7) | 120 | 15.9 |  | 0.87(0.68-1.11) |
| **Gender** |  |  | 0.716 |  |  |  | 0.755 |  |
| Male | 1104(84.9) | 1139(84.7) |  | 284(85.8) | 221 | 14.7 |  | 1 |
| Female | 196(15.1) | 205(15.3) |  | 47(14.2) | 37 | 12.7 |  | 1.06(0.75-1.50) |
| **Smoking status** |  |  | 0.549 |  |  |  | 0.192 |  |
| No | 526(40.5) | 572(42.6) |  | 120(36.3) | 90 | 16 |  | 1 |
| Yes | 774(59.5) | 772(57.4) |  | 211(63.7) | 168 | 13.5 |  | 1.19(0.92-1.53) |
| **Drinking status** |  |  | <0.001 |  |  |  | 0.006 |  |
| No | 536(41.2) | 742(55.2) |  | 127(38.4) | 94 | 18 |  | 1 |
| Yes | 764(58.8) | 602(44.8) |  | 204(61.6) | 164 | 12.6 |  | 1.43(1.12-1.84) |
| **BCLC stage** |  |  | -- |  |  |  | 0.389 |  |
| stage B | -- | -- |  | 304(91.8) | 236 | 14.5 |  | 1 |
| stage C | -- | -- |  | 27(8.2) | 22 | 13 |  | 1.21(0.78-1.88) |
| **Chemotherapy or TACE** |  |  | -- |  |  |  | <0.001 |  |
| None | -- | -- |  | 91(27.5) | 77 | 3.4 |  | 1 |
| Yes | -- | -- |  | 240(72.5) | 181 | 16.8 |  | 0.39(0.29-0.51) |

MST, median survival time; HR, hazard ratio; CI, conﬁdence interval; HCC, hepatocellular carcinoma; BCLC stage, Barcelona Clinic Liver Cancer stage; TACE, transcatheter hepatic arterial chemoembolization.

“-- “, garbage data in our analyses.

^a^ HCC patients who stay at stage B or stage C and without surgery.

Table S2. Stratified analyses on association between rs1076064 and risk of HCC

| **Variables** | **HCC patients / HBV carriers** | | | **Adjusted OR (95%CI) ^a^** | ***P* ^b^** |
| --- | --- | --- | --- | --- | --- |
|  | **AA** | **AG** | **GG** |  |  |
| **Age** |  |  |  |  |  |
| ≤53 | 189/181 | 332/315 | 166/199 | 0.88(0.76-1.02) | 0.836 |
| >53 | 189/174 | 266/295 | 149/168 | 0.90(0.78-1.06) |  |
| **Gender** |  |  |  |  |  |
| Male | 319/298 | 509/517 | 268/312 | 0.89(0.79-1.00) | 0.640 |
| Female | 59/57 | 89/93 | 47/55 | 0.96(0.71-1.28) |  |
| **Smoking status** |  |  |  |  |  |
| Ever | 237/212 | 367/350 | 167/201 | 0.87(0.75-1.01) | 0.493 |
| Never | 141/143 | 231/260 | 148/166 | 0.94(0.80-1.11) |  |
| **Drinking status** |  |  |  |  |  |
| Ever | 235/153 | 351/274 | 175/170 | 0.85(0.74-0.99) | 0.222 |
| Never | 143/202 | 247/336 | 140/197 | 0.97(0.83-1.13) |  |

CI, confidence interval; HBV, hepatitis B virus; HCC, hepatocellular carcinoma; OR, odds ratio.

^a^ Logistic regression analyses adjusted for age, gender, smoking status and drinking status in the additive genetic model (excluded the stratified factor in each stratum).

^b^ *P* for heterogeneity

Table S3. Stratified analysis of rs1076064 genotypes associated with HCC survival

| **Variables** | **rs1076064(deaths/patients)** | | | **Adjusted HR (95% CI)^a^** | ***P* ^b^** |
| --- | --- | --- | --- | --- | --- |
|  | **AA** | **AG** | **GG** |  |  |
| **Age** |  |  |  |  |  |
| <=53 | 33/40 | 74/88 | 30/44 | 0.71(0.55-0.91) | 0.813 |
| >53 | 42/51 | 52/66 | 26/41 | 0.68(0.53-0.88) |  |
| **Gender** |  |  |  |  |  |
| Male | 68/81 | 107/130 | 45/72 | 0.65(0.54-0.78) | 0.032 |
| Female | 7/10 | 19/24 | 11/13 | 1.23(0.71-2.14) |  |
| **Smoking status** |  |  |  |  |  |
| No | 21/26 | 46/60 | 22/33 | 0.84(0.62-1.15) | 0.207 |
| Yes | 54/65 | 80/94 | 34/52 | 0.66(0.53-0.81) |  |
| **Drinking status** |  |  |  |  |  |
| No | 27/33 | 48/62 | 18/31 | 0.73(0.54-1.00) | 1.000 |
| Yes | 48/58 | 78/92 | 38/54 | 0.73(0.59-0.90) |  |
| **Chemotherapy or TACE** |  |  |  |  |  |
| None | 26/28 | 35/41 | 16/22 | 0.74(0.54-1.01) | 0.889 |
| Yes | 49/63 | 91/113 | 40/63 | 0.76(0.62-0.93) |  |

HR, hazard ratio; CI, conﬁdence interval; HCC, hepatocellular carcinoma; TACE, transcatheter hepatic arterial chemoembolization.

^a^ Adjusted for age, gender, smoking status, drinking status, BCLC stage, and chemotherapy or TACE status(excluded the stratified factor in each stratum).

^b^ *P* for heterogeneity
